# Sociodemographic Determinants in Cervical Cancer Screening Among the Underserved West Texas Women

**DOI:** 10.1089/whr.2022.0050

**Published:** 2023-04-19

**Authors:** Brooke Jensen, Hafiz Khan, Rakhshanda Layeequr Rahman

**Affiliations:** ^1^ School of Medicine, Texas Tech University Health Sciences Center, Lubbock, Texas, USA.; ^2^ Julia Jones Matthews Department of Public Health, Texas Tech University Health Sciences Center, Lubbock, Texas, USA.; ^3^ Department of Surgery, Texas Tech University Health Sciences Center, Lubbock, Texas, USA.; ^4^ Southwest Cancer Center, University Medical Center Lubbock, Lubbock, Texas, USA

**Keywords:** cervical cancer, cancer screening, health disparities

## Abstract

**Objectives:**

Pap smear screenings are associated with a 60% reduction in cervical cancer rates for women over the age of 40 years. West Texas presents a challenge for cervical cancer screening as demonstrated by some of the highest incidence and mortality rates of any region in Texas. This study examined the role of socioeconomic and sociodemographic factors in the nonadherence of underserved/uninsured women treated by Access to Breast and Cervical Cancer Care for West Texas (ABC^2^4WT) in three regions with the goal of identifying barriers to screening and higher risk groups.

**Methods:**

ABC^2^4WT Program database was queried from November 1, 2018, to June 1, 2021, for sociodemographic variables, screening history, and screening results to identify high-risk groups for outreach. Independent samples *t*-test, Pearson's chi square test, and logistic regression were used to detect significant relationships between variables.

**Results:**

There were 1,998 women from the ABC^2^4WT Program included in the study. The program's rates of abnormal pap tests were 21.5% (Council of Government 1 [COG-1]), 8.1% (Council of Government 2 [COG-2]), and 9.6% (Council of Government 7 [COG-7]), all much higher than the nation's average of 5%. Women without recent cervical screening (5 or more years) represented 31.8% (*n* = 183) of COG-1, 40.3% (*n* = 132) of COG-2, and 49.5% (*n* = 61) of COG-7. In addition, a lower baseline adherence rate was noted in women with reduced incomes (<$600 per month per person) than those with higher incomes (*p* = 0.008). Non-Hispanic women were two times more likely to “no-show” screening appointments than Hispanic women (odds ratio [OR] = 2.01, 95% confidence interval [CI] 1.31–3.08). However, Hispanic women required two times more colposcopies and biopsies (OR = 2.08, 95% CI 1.05–4.13).

**Conclusions:**

Hispanic race and poverty represent a high-risk category for cervical cancer and form an important target for community outreach in West Texas.

## Introduction

The decrease in cervical cancer incidence and mortality over the past 40 years is attributed to regular pap screens initiated in the 1960s.^1^ The American College of Obstetricians and Gynecology (ACOG), Society for Colposcopy and Cervical Pathology (ASCCP), and Society of Gynecologic Oncology (SGO) have guidelines for cervical cancer screening. Based on these guidelines, women 21–29 years of age should receive cervical cancer screening every 3 years without high-risk human papillomavirus (hrHPV) and women 30–65 years of age should receive screening every 5 years with primary hrHPV testing alone or in combination with cytology (cotesting).^2,3^

Despite wide screening availability and advocacy efforts to enhance awareness for the prevention of cervical cancer in the United States, several barriers persist.^4^ The *Healthy People 2020* target for cervical cancer screening was set at 93%^5^; however, no group of women in the United States achieved this goal.^6^

Previous studies focused on delineating barriers to cervical screening, report sociodemographic variables, income levels, educational level, immigration status, and geographic variables as potential intervention points to increase screening rates.^7^ Evidence suggests that Hispanic women are less likely to be screened for cervical cancer compared with non-Hispanic women.^8^ In addition, women with lower educational levels are less likely to be screened and/or to follow-up on abnormal results. Access issues, such as lack of health insurance^9^ and low income,^10^ as well as social barriers, immigration,^11^ and refugee status,^12^ pose additional barriers for cervical screening adherence.

Moreover, lack of access and lower uptake of screening are well documented among rural counties compared with urban counties.^13^ Given the prevalence of these barriers in Texas,^14^ it is not surprising that Texas ranks 47th in the nation for pap smear adherence.^15^ Consequently, cervical cancer remains the third most common cancer diagnosed in Texan women aged 20–39 years and the fifth in women 40–49 years. The estimates for 2019 alone were 1,395 new cases and 447 deaths.^16^ Hispanic women had the highest cervical cancer incidence rate of 10.9 per 100,000 women, followed by non-Hispanic White women (9.6) and non-Hispanic Black women (8.7).^17^

**FIG. 1.**
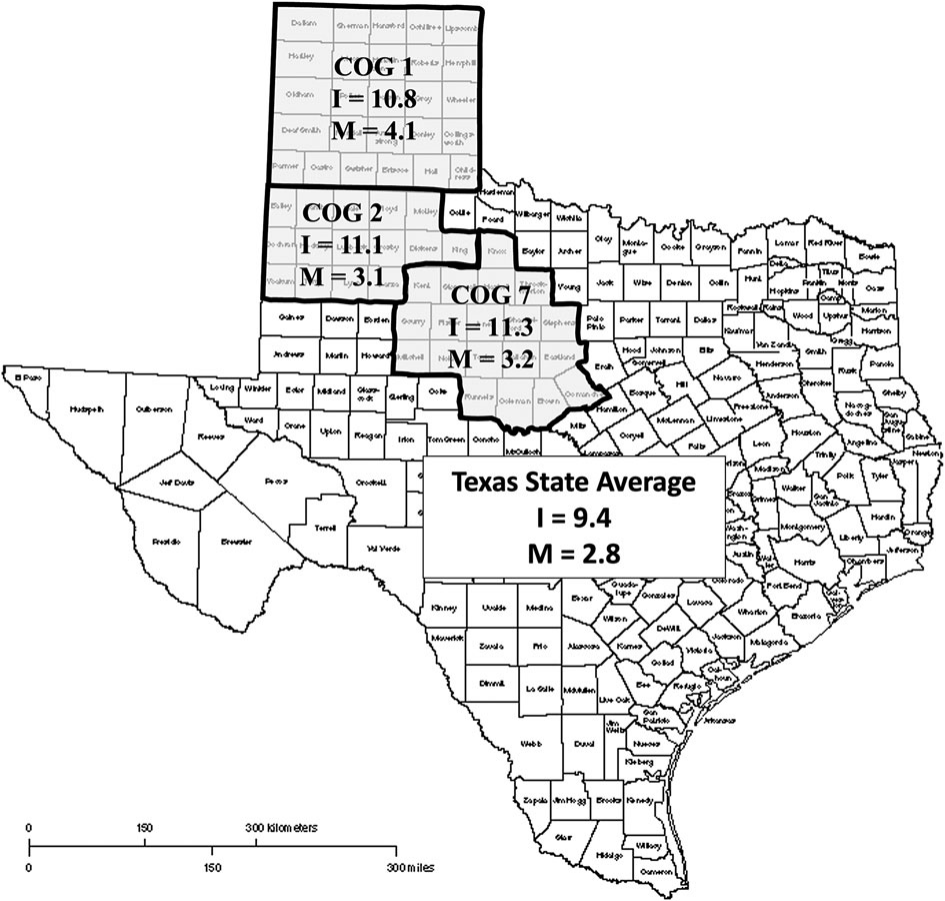
Target region for the ABC^2^4WT comprehensive community outreach project. Outlines mark the COG regions with age-adjusted incidence (I) and mortality (M) rates for cervical cancer per 100,000 women compared with the state averages from 2017 to 2019. ABC^2^4WT, Access to Breast and Cervical Cancer Care for West Texas; COG, Council of Government.

The basis for this study is the comprehensive community outreach program, Access to Breast and Cervical Cancer Care for West Texas (ABC^2^4WT), funded by Cancer Prevention and Research Institute of Texas (CPRIT). ABC^2^4WT is an outreach program intended for the underserved (uninsured/underinsured) population of West Texas. The Texas Panhandle, South Plains, and Central West Texas regions (Council of Government [COG] 1, 2, and 7, respectively) are the regions where ABC^2^4WT participants reside (Fig. 1).

In the years 2017 to 2019, the age-adjusted cervical cancer incidence rate (per 100,000 women) for all three COGs, COG-1 (10.8), COG-2 (11.1), and COG-7 (11.3), were far above the state average of 9.4. The age-adjusted mortality rates (per 100,000 women) in COG-1 (4.1), COG-2 (3.1), and COG-7 (3.2) also exceeded the state average of 2.8 (Fig. 1).^18^ Thus, cervical cancer screening has remained a priority for the CPRIT prevention program since its inception.^19^

Although cervical cancer screening is an invaluable tool that can detect cervical cancer in its early stages, the lack of adherence and access to these resources are a significant contributor to the high mortality and morbidity rates in this population. This study examined the role of socioeconomic and sociodemographic factors in the nonadherence of underserved/uninsured women treated by ABC^2^4WT in three regions (COG-1, COG-2, and COG-7) with the goal of identifying barriers to screening and higher risk groups.

## Methods

### Program description

ABC^2^4WT is an outreach program that represents a public–private-community partnership with the goal of increasing breast and cervical cancer screening rates in targeted regions. The focus of this study is cervical cancer screening using the American College of Obstetrics and Gynecology (ACOG) guidelines. These guidelines are how ABC^2^4WT determined the screening eligibility of the women who applied to their program.

These guidelines recommend pap smears for women aged 21–29 years every 3 years and cotesting for human papilloma virus (HPV) for women aged 30–65 years of age every 5 years.^20^ The program uses mass media campaigns and region-focused events for outreach in West Texas counties (counties within COG-1, COG-2, and COG-7). These campaigns and events are conducted by trained community outreach specialists to increase awareness and education about cervical cancer, as well as to offer no cost screening to women who are eligible by ACOG guidelines.

### Variables

The ABC^2^4WT outreach program maintains a prospective database for all participants served by the project. Data were collected on sociodemographic variables, health insurance status, and screening history through program applications completed by participants. The data collected between November 1, 2018, and June 1, 2021, were retrospectively analyzed for trends and associations within the population. The study population was sufficient to detect a statistically significant relationship assuming the level of significance, alpha (*α*) = 0.05.

Three main outcome measures were analyzed: (1) baseline adherence rate, defined as at least one pap smear in the past 5 years for age-eligible women (26 years and older, since screening per ACOG guidelines is not recommended until 21 years of age), (2) no-show rate, defined as women who failed to keep their current pap smear appointments after three attempts at scheduling, and (3) abnormal pap smear rate, defined as high-risk outcomes of pap smears. Independent variables analyzed for trend analysis included age, race/ethnicity, monthly income, number of persons on income, status of health insurance, county of residence, and results of prior testing if applicable.

### Statistical analysis

Basic descriptive measures (*e.g.*, means, standard deviations [SDs], ranges, histograms, and scatter plots) were obtained to determine distributions of patient characteristics. SPSS (Statistical Package for the Social Sciences) software version 27 was used to perform transformations, collapse categories, and analyze differences. Differences in patient characteristics were analyzed using appropriate test statistics (*t*-tests for continuous and Pearson's chi square test for categorical level data).

Analyses to evaluate the differences in pap smear outcomes and adherence rates by demographic and socioeconomic variables (age, monthly income, race/ethnicity, and county of residence within COG regions in Texas) were performed at both the bivariate and multivariate levels. First, unadjusted odds ratios (ORs) and 95% confidence intervals (CIs) were reported. A multivariable logistic regression was used to obtain the adjusted odds ratios (AORs) and the corresponding 95% CI. To provide a parsimonious model, only those covariates that are significant at the bivariate level were included in the multivariable logistic regression model.

### Statement of ethics and informed consent

This application was screened for exempt status according to TTUHSC policies and the provisions of applicable federal regulations (IRB No.: L21-191).

## Results

### Demographics

**FIG. 2.**
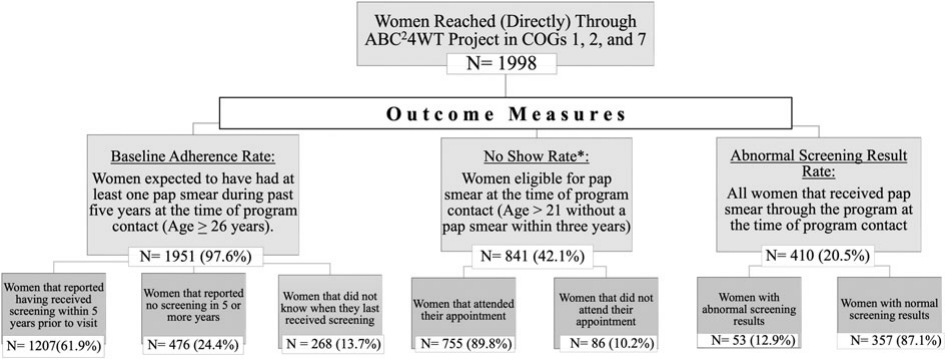
Flow diagram showing the distribution of participants and outcome measures.

The program reached 1,998 women from three COGs (COGs 1, 2, and 7) covered by the ABC^2^4WT project. Characteristics of the study sample organized by outcome variable are presented in Figure 2. One thousand nine hundred fifty-one (97.6%) participants were above the age of 26 years and thus expected to have had at least one pap smear within past 5 years from time of contact and 841 (42.0%) women were eligible for a pap smear at the time of the encounter per ACOG guidelines.

The mean (±SD) monthly income and age of ABC^2^4WT participants were $730 (±533) per month per person and 48.2 (±9.8) years, respectively. Of the 1,998 women reached by ABC^2^4WT, 65.5% were Hispanic (*n* = 1307), 28.4% were White (*n* = 566), and 4.5% were Black (*n* = 90). There were 854 (42.7%) participants in COG-1, 944 (47.3%) in COG-2, and 200 (10.0%) in COG-7.

The Texas Department of Housing and Community Affairs categorizes Texas counties as rural and urban; based on their regulations and recommendations, 11.4% (*n* = 228) of participants resided in rural areas, 85.9% (*n* = 1,714) in urban areas, and 2.7% (*n* = 53) in undefined areas and thus categorized as “other.”^21^ Over 90% (*n* = 1,811) of women included in this study were uninsured at the time of contact.

### Baseline adherence rate

According to the ACOG guidelines, women who are 26 years or older at program contact should be expected to have undergone at least one pap smear. One thousand nine hundred fifty-one women seen by the program were 26 years or more at time of contact and met the age criteria. The overall adherence rate for this group was 61.9% (*n* = 1,207); age groups, race, household income per person, and geographical criteria were analyzed as confounding variables (Table 1).

**Table 1.** Factors Associated with Baseline Compliance with Pap Smear Screening According to American College of Obstetrics and Gynecology Guidelines

Characteristics	Last Pap smear completed						Total participants in subgroup, ***n*** (%)	***p***
	<5 Years (adherent)		≥5 Years or never (nonadherent)		Missing or “Don't Know”			
	***n***	%	***n***	%	***n***	%		
Age (years)								
26–30	23	39.7	18	31.0	17	29.3	58 (3.0)	0.054
31–40	98	41.0	70	29.3	71	29.7	239 (12.3)	
41–50	394	44.4	188	21.2	305	34.4	887 (45.5)	
51–65	302	39.4	200	26.1	265	34.6	767 (39.3)	
Household income ($ per person per month)								
0–600	176	37.2	139	29.4	158	33.4	472 (29.8)	0.008
601+	641	43.4	337	22.8	500	33.8	1478 (75.8)	
Settlement								
Rural	86	38.1	54	23.9	86	38.1	226 (11.6)	0.617
Urban	708	42.3	411	24.6	554	33.1	1673 (85.5)	
Other	23	44.2	11	21.2	18	34.6	34 (2)	
Race								
White	191	34.5	159	28.8	203	36.7	553 (28.3)	<0.001
African American	35	44.3	16	20.3	28	35.4	79 (4.0)	
Hispanic	584	45.3	289	22.4	417	32.3	1290 (66.1)	
Texas region^*^								
COG-1	411	49.5	183	22.0	236	28.4	594 (49.5%)	<0.001
COG-2	344	36.8	232	24.8	358	38.3	576 (44.5)	
COG-7	62	33.2	61	32.6	64	34.2	123 (9.5)	

^*^ COG, Council of Government.

Baseline adherence rate was lowest in White women (34.5%) than in Black women (44.3%) and Hispanic women (45.3%) (*p* < 0.001). Women between the ages of 26 and 30 years at the time of contact had the lowest baseline adherence rate of any age group (39.7%, *p* = 0.054). In addition, women with a household income of <$600 per month per person had reduced baseline adherence at 37.2% than those with higher incomes (43.4%) (*p* = 0.008). Interestingly, there was a significant difference in regional baseline adherence; participants living in COG-7 had the lowest rate of baseline adherence (33.2%) when compared with other COGs (COG-1 [49.5%] and COG-2 [36.8%]; *p* < 0.001).

Multivariate analysis demonstrated several significant sociodemographic variable relationships with baseline nonadherence: COG-1, Black ethnicity, Hispanic ethnicity, ages 41–50 years, and incomes <$600 per month per person. The likelihood of participants presenting as baseline nonadherent was decreased in COG-1 (AOR = 0.522, 95% CI 0.344–0.790), Black ethnicity (AOR = 0.226, 95% CI 0.074–0.688), Hispanic ethnicity (AOR = 0.294, 95% CI 0.114–0.760), and ages 41–50 years (AOR = 0.734, 95% CI 0.570–0.947). Alternatively, there was an increased likelihood of participants with incomes <$600 per month per person presenting as baseline nonadherent (AOR = 1.515, 95% CI 1.164–1.971). No significant associations with settlement were observed on multivariate analysis.

### “No-show” rate

Eight hundred forty-one women were eligible for cervical screening at the time of participation in the ABC^2^4WT program. Despite three attempts at rescheduling, 10.2% (*n* = 86) of these women did not keep their appointment for screening pap smears and was deemed “no shows” (Table 2). The eligible participants were distributed across all three COGs with 42.6% (*n* = 358) originating from COG-1, 43.3% (*n* = 364) from COG-2, and 14.1% (*n* = 119) from COG-7.

**Table 2.** Factors Associated with “No-Show” Rate for Pap Smear Screening Appointments

Characteristics	No show for pap screening		Total participants in subgroup, ***n*** (%)	***p***
	***n***	%		
Age (years)				
≤30	11	13.3	64 (13.4)	0.676
31–40	17	12.2	106 (22.1)	
41–50	31	9.7	157 (32.8)	
≥50	27	9.0	152 (31.8)	
Household income ($ per month per person)				
0–600	12	5.5	218 (26.9)	0.082
601+	54	9.1	591 (73.1)	
Settlement				
Rural	13	16.0	81 (9.6)	0.041
Urban	73	9.8	743 (88.3)	
Other	0	0.0	17 (2.0)	
Race				
White	37	13.9	267 (31.7)	0.015
African American	2	4.7	43 (5.1)	
Hispanic	43	8.4	514 (61.1)	
Texas region^*^				
COG-1	35	9.8	358 (42.6)	< 0.001
COG-2	23	6.3	364 (43.3)	
COG-7	28	23.5	119 (14.1)	

^*^ COG, Council of Government.

A significant percentage of the eligible participants for screening lived in urban settlements (88.3%) versus rural settlements (9.6%) or other areas (2.0%) (*p* = 0.023). Screen-eligible women ranged from 21 years to >65 years with the most prevalent age group being those from 41 to 50 years of age (35.9%). Race/ethnicity demonstrated significant variation in eligibility for screening with Hispanics having the lowest percentage of women eligible for pap smear at the time of contact (39.3%).

Among participants eligible for screening, White women (13.9%) were found to have the highest “no-show” rate compared with Black (4.7%) and Hispanic (8.4%) women (*p* = 0.015). Rural participants had a “no-show” rate of 16% compared with 9.8% among urban participants (*p* = 0.041). Women with household incomes <$600 per month per person had a lower “no-show” rate (5.5%) than women with higher incomes (9.1%) (*p* = 0.082). Participants living in COG-7 had the highest rate of “no show” (23.5%) when compared with those living in COG-1 (9.8%) and COG-2 (6.3%) (*p* < 0.001).

Multivariate analysis demonstrated three significant sociodemographic variable relationships with “no-show” rate: COG-1, COG-2, and baseline nonadherence. The likelihood of “no-show” participants was decreased in COG-1 (AOR = 0.332, 95% CI 0.185–0.595) and COG-2 (AOR = 0.202, 95% CI 0.108–0.380). Participants with baseline nonadherence were more likely to “no show” (AOR = 1.718, 95% CI 0.978–3.020). No significant associations with age, settlement, or income were observed on multivariate analysis.

### Abnormal pap smears

Four hundred ten women received “no cost” cervical screening at the time of the contact; 53 of these women (12.9%) had an abnormal screening result. Abnormal results seen in this group encompassed atypical squamous cells of undetermined significance (ASC-US), atypical squamous cells, cannot exclude high-grade intraepithelial lesion (ASC-H), atypical glandular cells, low-grade squamous intraepithelial lesions, high-grade squamous intraepithelial lesions, and carcinoma.

Of the 53 (12.9%) women with “abnormal” cervical screens, 47 (11.4%) women were referred for further diagnostic testing for “high-risk lesions” *via* biopsy or colposcopy. Factors associated with abnormal pap smears are depicted in Table 3. The mean monthly gross income was not statistically significant when comparing participants who received “normal screening results” ($909 per month per person) and those who received abnormal results ($566 per month per person) (*p* = 0.184). The mean (±SD) age for normal screening was 44.6 (±10.9) years versus 41.3 (±10.5) years for abnormal screening with borderline significance (*p* = 0.043).

**Table 3.** Factors Associated with “Abnormal Pap Screening Results”

Characteristics	Pap screening outcomes						Total participants in subgroup, ***n*** (%)	***p***
	No lesions (NILM)		Low-risk lesions (ACS-US, LSIL)^*^		High-risk lesions (ACS-H, AGC, HSIL, carcinoma)^*^			
	***n***	%	***n***	%	***n***	%		
Age (years)								
≤30	47	85.5	5	9.1	3	5.5	55 (13.4)	0.551
31–40	73	82.0	8	9.0	8	9.0	89 (21.7)	
41–50	119	88.1	12	8.9	4	3.0	135 (32.9)	
≥50	118	90.1	9	6.9	4	3.0	131 (32.0)	
Household income ($ per person per month)								
0–600	77	88.5	8	9.2	2	2.3	87 (22.3)	0.482
601+	263	86.8	24	7.9	16	5.3	303 (77.7)	
Settlement								
Rural	27	96.4	1	3.6	0	0.0	28 (6.8)	0.503
Urban	325	86.2	33	8.8	19	5.0	377 (92.0)	
Other	5	100	0	0.0	0	0.0	5 (1.2)	
Race								
White	119	90.2	10	7.6	3	2.3	132 (32.6)	0.133
African American	25	96.2	1	3.8	0	0.0	26 (6.4)	
Hispanic	209	84.6	23	9.3	15	6.1	247 (61.0)	
Texas region^*^								
COG-1	136	79.5	18	10.5	17	9.9	171 (41.7)	<0.001
COG-2	157	92.9	11	6.5	1	0.6	169 (41.2)	
COG-7	64	91.4	5	7.1	1	1.4	70 (17.1)	
Last pap screening								
<5 Years	145	82.2	15	9.4	5	8.3	165 (40.2)	0.015
≥5 Years	147	91.7	10	7.1	4	2.5	161 (39.5)	
Missing or “Don't Know”	65	77.4	9	10.7	10	11.9	84 (20.5)	

^*^ COG, Council of Government.

ACS, atypical squamous cells; ACS-H, atypical squamous cells, cannot exclude high-grade intraepithelial lesion; ACS-US, atypical squamous cells of undetermined significance; AGC, atypical glandular cells; HSIL, high-grade squamous intraepithelial lesions; LSIL, low-grade squamous intraepithelial lesions; NILM, negative for intraepithelial lesion or malignancy.

Other significant factors for abnormal results were residence in COG-1 (20.4% abnormal; *p* < 0.001) and Hispanic ethnicity (15.0%, *p* = 0.048). Women who choose to not report their last pap smear date or listed “don't know” were most likely to have abnormal findings (22.6%; *p* = 0.015). Hispanic women were 1.8 times more likely to have an abnormal screening and 2.6 times more likely to require a biopsy or colposcopy than non-Hispanic women (OR = 1.808, 95% CI 0.943–3.466; OR = 2.591, 95% CI 1.292–5.197). Multivariate analysis revealed that residence in COG-1 and “unknown/unreported” pap screening dates was significantly associated with abnormal pap smear results (AOR = 4.482, 95% CI 1.304–15.409; AOR = 2.330, 95% CI 1.091–4.980, respectively).

### Baseline nonadherence and “no-show” status

Previously nonadherent participants were subdivided into two groups, those who attended their contemporary pap screening appointments and those who did not. Fifty-six (11.8%) of the baseline nonadherent women (*n* = 476) did not attend their appointment despite at least three attempts for rescheduling and were deemed as “no show.” Factors associated with this subgroup of nonadherent women are depicted in Table 4. Previously nonadherent Black women had 100% appointment attendance and were the most likely to attend their appointments compared with Hispanic (89.6%) and White women (84.9%) (*p* = 0.047).

**Table 4.** Factors Associated with Nonadherence and “No-Show” Rate for Pap Smear Screening Appointments

Characteristics	Nonadherent (>5 years) and “No-Show” participants		Total participants in subgroup, ***n*** (%)	***p***
	***n***	%		
Age (Years)				
26–30	2	11.1	18 (3.8)	0.729
31–40	11	15.7	70 (14.7)	
41–50	20	10.6	188 (39.5)	
≥50	23	11.5	200(42.0)	
Household income ($ per person per month)				
0–600	11	7.9	139 (29.2)	0.094
601+	45	13.4	337 (70.8)	
Settlement				
Rural	8	14.8	54 (11.3)	0.201
Urban	48	11.7	411 (86.3)	
Other	0	0.0	11 (2.3)	
Race				
White	24	15.1	159 (34.3)	0.047
African American	0	0.0	16 (3.4)	
Hispanic	30	10.4	289 (62.3)	
Texas region^*^				
COG-1	21	11.5	183 (38.4)	<0.001
COG-2	18	7.8	232 (48.7)	
COG-7	17	27.9	61 (12.8)	

^*^ COG, Council of Government.

Multivariate analysis demonstrated significant associations with COG-1, COG-2, and income <$600 per month per person for “no-show” rates in previously nonadherent participants. The associations observed showed decreased “no-show” rates for COG-1 (AOR = 0.330, 95% CI 0.160–0.682), COG-2 (AOR = 0.187, 95% CI 0.088–0.400), and income of <$600 per month per person (AOR = 0.544, 95% CI 0.267–1.107). No significant associations with age, settlement, or ethnicity were observed on multivariate analysis.

## Discussion

This study was designed to identify potential barriers to cervical cancer screening among underserved women of West Texas. Since ABC^2^4WT is primarily aimed toward underserved women, the program's population represents a known high-risk group for cancer screening in terms of nonadherence. This analysis provides a more granular assessment of risk factors within this population, which will aid with strategic planning for similar targeted outreach programs.

In this largely uninsured population, we found the lowest rate of adherence among White women than among ethnic minorities; interestingly, this finding is consistent with older data documenting Black women having higher cervical cancer screening rates than White women for over a decade.^22^ More recent data collected through Qualtrics online surveys report lower rate of pap screening adherence among Black women.^4^

In previous studies, younger women have been described as more adherent with screening than older women, which is the opposite of our experience with this study.^23^ In addition, women with no history of screening were unlikely to identify personal barriers, structural barriers (transportation, work excuses, *etc*.), and cultural barriers as limiting factors for screening;^9^ however, the variation in our study's baseline adherence rates by region, settlement, and income suggests that both cultural and structural barriers have limited these participant groups' initial cervical cancer screenings.^24^

Participants living in COG-7 and/or urban settlements had the lowest rates of baseline adherence that can be attributed to a combination of cultural and structural barriers in these areas. Rural settlement is often found to be a barrier for equitable delivery of health care; however, University of Washington Rural Health Research Center study reported that whereas mammographic screening improved over a span of 11 years but rural women remained less likely to receive mammograms, pap smear screening did not reveal any temporal change or differences between urban and rural settings.^23^ These observations might point to the cultural barriers being more profound than the structural barriers for cervical cancer screening.

As in previous studies, this study found that access factors and structural barriers, such as health insurance, transportation, and cost, are important barriers to cervical cancer screening and were found to limit regular screenings.^9,25,26^ Our results demonstrated that women with lower household incomes were found to have a significantly lower rate of baseline adherence relative to those with larger household incomes. When removing structural barriers and improving access (*i.e.*, providing no cost screenings), women with household incomes <$600 per month per person were more likely to attend screening appointments than women with higher incomes.^24^

Among the participants eligible for screening, White women demonstrated the highest “no-show” rate compared with Black and Hispanic women. These results are congruent with the Behavioral Risk Factor Surveillance Survey (BRFSS) that determined that Hispanic and Black women were more likely to receive a recent pap smear than White women.^27^ Owing to high baseline adherence, fewer women in rural settlements were deemed eligible for cervical screens; thus, the participants with “no show” were likely nonadherent individuals due to personal or cultural barriers rather than an issue with access to care.

These “no-show” individuals may lack adequate screening education and awareness, thus causing their nonadherence. A limitation of our study is that no information on education level or awareness of guidelines was collected, which makes it difficult to ascertain whether lack of knowledge played a role in missing screening appointments.^4^ Johnson et al reported that there was no significant association between knowledge and screening behavior among populations that were sampled to represent groups with high rate of cervical cancer (20% Blacks, 20% Hispanics, and 20% with annual income of <$30,000).

Another curious finding in our study is that COG-7 (Central West Texas) had the highest rate of “no show” when compared with COG-1 (Texas Panhandle) and COG-2 (South Plains Texas). Since ABC^2^4WT addressed the structural barriers of cost (free services and work excuses), and transportation (travel vouchers), there is potential that this discrepancy results from cultural differences between regions. Another potential barrier for participants is community trust in the program and its providers as COG-7 is the newest and thus least established region to participate in the program. This insight is valuable to develop strategies and direct resources more focused on overcoming the nonstructural cultural barriers in this region.

Cervical cancer screening adherence has been reportedly higher among younger women, thus, making early stage detection of cervical cancer more likely.^28^ The mean age for abnormal screening results in our analysis was 41.3 years, which was significantly lower than the mean for normal screenings at 44.6 years. Thus, older participants in this study were more likely to receive normal screenings contrary to previously reported statistics.

Multivariate analysis revealed that residence in COG-1 (Texas Panhandle) and “unknown/unreported” pap screening dates were also significantly associated with abnormal pap smear results. Interestingly, COG-1 is the region with the highest participant adherence rate as well as the highest rate of abnormal screens. Since we did not collect data on behavioral risk factors, it is difficult to determine whether the high adherence rate is the reason for higher rate of abnormal results or other behavioral factors prevail in the region that might be associated with high rate of cervical dysplasia.

Participants of Hispanic ethnicity were 1.8 times more likely to have an abnormal screening and 2.6 times more likely to require a biopsy or colposcopy than non-Hispanic women. These findings are consistent with prior study results demonstrating that Hispanic women have higher odds of having advanced invasive cervical cancer than non-Hispanics at the time of diagnosis.^29^ Despite household income impacting both baseline adherence and “no-show” rates, this risk factor was not statistically significant for abnormal screening results.

The Black population percentage in West Texas is lower than in metropolitan areas of Texas (average 12.7%),^30^ ranging from 1.1% to 10.1% of the total West Texas population; therefore, these findings may be not generalizable to regions with different demographics. Previously nonadherent Black women had 100% appointment attendance after the removal of structural barriers. Health insurance, transportation, and work excuses were thus the limiting factor for cervical cancer screenings in this group of West Texas participants. Previously nonadherent Hispanic and White women were less likely to attend appointments despite accounting for region, income, and age.

### Strengths and limitations of the study

The strengths of this study lie in the extended coverage of three different COG regions encompassing 60 counties; however, not all counties in the target regions were reached by the program. The study was limited in the inferences made with regard to COG-7 since it is the smallest sampled region in terms of population. An extended study period of COG-7 could decrease the need for conjecture and improve statistical CIs. In addition, no data on HPV vaccinations or rates, educational level, guideline awareness, cultural beliefs, religious beliefs, or behavioral risk factors were obtained through this program. Therefore, the association of adherence or “no-show” rate with these factors could not be analyzed.

Some cultural barriers seen by these counties were able to be addressed by a specific liaison hired from the intended region with the purpose of better understanding individuals from the area. Owing to the many locations encompassed by the program, not all cultural barriers could be addressed.

## Conclusions

Underserved women of West Texas mainly comprise Hispanics and Whites. Poverty is the main structural barrier with lowest adherence associated with household income of <$600 per person per month. However, addressing this barrier is still associated with significant “no-show” rate most likely due to cultural and other nonstructural barriers. Longevity of the program tends to increase adherence through mature public–private-community partnerships.

To improve and mature community programs, regions with high screening rates should be further analyzed and potentially partner with regions demonstrating low screening rates. An understanding of these unique local factors can enable health care providers and the public health workers to develop innovative strategies to increase screening of cervical cancer. Ultimately, Texas needs to focus on developing strategies that identify high-risk groups based on income, Hispanic race or ethnicity, older women, region of service, and length between screenings.

## Abbreviations Used

ABC^2^4WT: Access to Breast and Cervical Cancer Care for West Texas
ACOG: American College of Obstetricians and Gynecology
AOR: adjusted odds ratio
ASCCP: Society for Colposcopy and Cervical Pathology
ASC-H: atypical squamous cells, cannot exclude high-grade intraepithelial lesion
ASC-US: atypical squamous cells of undetermined significance
BRFSS: Behavioral Risk Factor Surveillance Survey
CI: confidence interval
COG: Council of Government
CPRIT: Cancer Prevention and Research Institute of Texas
HPV: human papilloma virus
hrHPV: high-risk human papillomavirus
HSIL: high-grade squamous intraepithelial lesions
LSIL: low-grade squamous intraepithelial lesions
OR: odds ratio
SD: standard deviation
